# Fungal infections in adult patients on extracorporeal life support

**DOI:** 10.1186/s13054-018-2023-z

**Published:** 2018-04-17

**Authors:** Yiorgos Alexandros Cavayas, Hakeem Yusuff, Richard Porter

**Affiliations:** 1 Département de Soins Critiques, Hôpital Sacré-Coeur de Montréal, 5400 Boul Gouin Ouest, Montreal, QC H4J 1C5 Canada; 2 University Hospitals of Leicester, ECMO program, Glenfield Hospital, Groby Rd, Leicester, LE3 9QP UK

**Keywords:** Extracorporeal membrane oxygenation [D015199], Invasive fungal infections [D000072742], *Aspergillus* [D001230], Aspergillosis [D001228], *Candida* [D002175], Candidemia [D058387]

## Abstract

**Background:**

Patients on extracorporeal membrane oxygenation (ECMO) are often among the most severely ill in the intensive care unit. They are often receiving broad-spectrum antibiotics; they have multiple entry points for pathogens; and their immune system is impaired by blood circuit interaction. These factors are thought to predispose them to fungal infections. We thus aimed to evaluate the prevalence, risk factors, and prognosis of fungal infections in adults on ECMO.

**Methods:**

We conducted a retrospective cohort study using the Extracorporeal Life Support Organization registry, which compiles data on ECMO use from hundreds of international centers. We included all adult patients from 2006 to 2016 on any mode of ECMO with either a diagnosis of fungal infection or a positive fungal culture.

**Results:**

Our study comprised 2129 adult patients (10.8%) with fungal colonization or infection. *Aspergillus* involvement (colonization or infection) was present in 272 patients (1.4%), of whom 35.7% survived to hospital discharge. There were 245 patients (1.2%) with *Candida* invasive bloodstream infection, with 35.9% survival. Risk factors for *Aspergillus* involvement included solid organ transplant (OR 1.83; *p* = 0.008), respiratory support (OR 2.75; *p* < 0.001), and influenza infection (OR 2.48; *p* < 0.001)*.* Risk factors for candidemia included sepsis (OR 1.60; *p* = 0.005) and renal replacement therapy (OR 1.55; *p* = 0.007). In multivariable analysis, *Aspergillus* involvement (OR 0.40; *p* < 0.001) and candidemia (OR 0.47; *p* < 0.001) were both independently associated with decreased survival.

**Conclusions:**

The prevalence of *Aspergillus* involvement and *Candida* invasive bloodstream infection were not higher in patients on ECMO than what has been reported in the general intensive care population. Both were independently associated with a reduced survival. *Aspergillus* involvement was strongly associated with ECMO for respiratory support and influenza.

**Electronic supplementary material:**

The online version of this article (10.1186/s13054-018-2023-z) contains supplementary material, which is available to authorized users.

## Background

Critically ill patients are at increased risk of fungal infections. *Candida* and *Aspergillus* are the most frequently isolated fungi in the intensive care unit (ICU). In fact, *Candida* is the third most prevalent microorganism in patients with infections the ICU [1]. It is responsible for 12% of ICU-acquired bloodstream infections [2]. Nosocomial *Candida* bloodstream infection (CBSI) is associated with increased morbidity and mortality as well as a prolonged hospital length of stay compared with other bloodstream infections [3]. Crude mortalities of 40–60% and attributable mortalities of 5–71% have been reported in patients with *Candida* infection in the ICU [4–6]. Invasive aspergillosis (IA) has typically been described in patients with hematological malignancies and profound immunosuppression, but recent evidence has suggested that it may be present in a significant proportion of critically ill patients without these conventional risk factors [7]. Researchers in previous studies have reported a 1–2% prevalence of *Aspergillus* [8–11] in the respiratory tract of mechanically ventilated patients and up to 8% in those with acute respiratory distress syndrome (ARDS) [12]. In an autopsy series of patients who died of ARDS, a prevalence of 12.5% was described [13]. Even in the absence of hematological malignancy, isolation of *Aspergillus* in critically ill patients has been associated with mortality as high as 86% [14].

In the last decade, use of extracorporeal membrane oxygenation (ECMO) has significantly increased in the adult population [15]. It is now routinely used to support critically ill patients with severe respiratory or cardiac failure in whom conventional therapies have failed. The severity of their illness and blood circuit interactions are thought to impair their immune system. They have catheters, cannulas, and oxygenators that can become colonized with microorganisms. They are often treated with broad-spectrum antibacterial agents. These factors are thought to predispose them to fungal colonization and infection. Recently reported data suggest that up to 15% of all bloodstream infections while on ECMO are of fungal origin [16]. A 6.4% prevalence of CBSI in an Australian center has been reported [17]. In another study, yeast DNA was retrieved in 7% of oxygenators after weaning [18].

All of these studies were performed in single centers. Fungal infection rates can vary greatly between units, depending on the type of patients treated and differences in local antibiotic and antifungal prescription practices. Robust multicenter data are lacking. Therefore, the objective of the present study was to establish the prevalence, risk factors, and prognosis of fungal infection and colonization in an adult ECMO population composed of a large international cohort of patients.

## Methods

### Study design

We performed a retrospective cohort study using data from the Extracorporeal Life Support Organization (ELSO) registry. The registry compiles data on ECMO use in more than 300 international centers after approval by local institutional review boards. For each ECMO run, participating centers complete a standardized data sheet containing patient demographics, diagnosis and procedure information, ECMO technique, physiological and microbiological data, complications, and outcomes (*see* Additional file 1: Appendix 1). After approval by the ELSO Registry Committee, limited de-identified datasets are released to participating centers for research purposes without the need for further approval from individual centers. This study was conducted in accordance with the amended Declaration of Helsinki.

### Population

We included all consecutive adult patients with fungal infection or colonization from January 2006 to September 2016 on any mode of extracorporeal life support. These patients were identified as those having either a diagnosis of fungal infection according to the International Classification of Diseases, Ninth Revision, or any positive fungal culture (*see* Additional file 1: Appendix 2).

### Statistical analysis

Analyses were performed using IBM SPSS Statistics for Mac software version 24.0 (IBM, Armonk, NY, USA). Categorical variables were summarized using frequencies and percentages, and continuous variables were summarized using mean and SD. Pearson’s chi-square test was used to test for univariate associations of categorical variables, and the Mann-Whitney *U* test was used for continuous variables. Missing variables were excluded from analyses.

To determine factors independently associated with *Aspergillus* involvement (colonization or infection) and *Candida* bloodstream infection, we performed a multivariable logistic regression using a stepwise backward selection model with a *p* value less than 0.20 for inclusion and greater than 0.15 for exclusion. In order to determine if *Aspergillus* involvement and CBSI were independently associated with a lower survival rate, we also used a multivariable logistic regression with a stepwise backward selection model with the same conditions for inclusion and exclusion. All of the models were validated by bootstrapping with 1000 samples. The variables entered into the models can be found in Additional file 1: Appendixes 5–7.

## Results

**Fig. 1:**
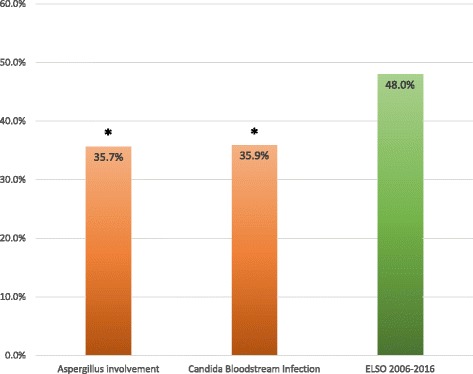
Survival of patients with *Aspergillus* involvement and *Candida* bloodstream infection compared with the overall survival in the Extracorporeal Life Support Organization (ELSO) registry during study the period. *Statistically significant difference compared with overall survival in the ELSO registry during the study period

There were 19,697 adult patients in ELSO registry during the study period. Of these, 2129 had either a diagnosis of fungal infection or a positive fungal culture, resulting in a prevalence of 10.8% (95% CI 10.4–11.2%) of infection and colonization. Their mean age was 48.5 years. The majority (67.3%) of patients received ECMO for respiratory support, and a majority (57.8%) were on venovenous ECMO (Table 1). The overall survival of patients with fungus did not significantly differ from the survival of those without (49.7% vs 48.7%; *p* = 0.559). However, two main subgroups emerged as having a different survival in univariate analyses: patients with an aspergillosis diagnosis or any *Aspergillus*-positive culture (35.7%; *p* < 0.001) and those with *Candida* in blood culture (35.9%; *p* < 0.001) (Fig. 1). There were 272 patients with *Aspergillus* involvement in the registry, which resulted in a prevalence of 1.4% (95% CI 1.2–1.5%), and 245 patients with CBSI, which resulted in a prevalence of 1.4% 1.2% (95% CI 1.1–1.4%) (Table 2). Positive *Candida* cultures in sites other than blood did not affect survival, no matter how many sites were affected (*see* Additional file 1: Appendix 4).

**Table 1:** Characteristics of patients with fungal colonization or infection

Variable	Summary (*N* = 2129)	
Age, years, mean ± SD	48.5 ± 15.7	
Sex, male, *n* (%)	1264	(59.7%)
Weight, kg, mean ± SD	85.1 ± 26.7	
Support type, *n* (%)		
Respiratory	1433	(67.3%)
Cardiac	563	(26.4%)
ECPR	133	(6.2%)
ECMO configuration, *n* (%)		
VA only	742	(34.8%)
VV only	1231	(57.8%)
Hybrid or conversion	134	(6.3%)
Sepsis, *n* (%)	505	(23.7%)
Pneumonia, *n* (%)	464	(21.8%)
Influenza, *n* (%)	301	(14.1%)
Acute respiratory distress syndrome, *n* (%)	297	(14.0%)
Other acute respiratory failure, *n* (%)	711	(33.4%)
Heart failure, *n* (%)	554	(26.0%)

*Abbreviations: ECPR* Extracorporeal cardiopulmonary resuscitation, *VA* Venoarterial, *VV* Venovenous

**Table 2:** Prevalence and outcome of colonization and fungal infections

Fungus/diagnosis	Total	Prevalence	Survival	Survivors		Nonsurvivors		
	No.	%	%	No.	%	No.	%	Chi-square *p* value
Diagnosis of aspergillosis	69	0.4%	28%	19	1.8%	50	4.7%	0.003
Culture of *Aspergillus* without diagnosis	203	1.0%	38%	78	7.4%	125	12%	0.009
*Aspergillus* (all)	272	1.4%	36%	97	9.2%	175	16%	< 0.001
Diagnosis of systemic candidiasis	27	0.1%	44%	12	1.1%	15	1.4%	0.959
Culture of *Candida* in blood	245	1.2%	36%	88	8.3%	157	15%	< 0.001
Culture of *Candida* in respiratory tract	1254	6.4%	55%	688	65%	566	53%	< 0.001
Culture of *Candida* in urine	295	1.5%	53%	155	15%	140	13%	0.783
*Candida* (all)	1907	9.7%	51%	977	92%	930	87%	0.001
Diagnosis of blastomycosis	17	0.1%	53%	9	0.8%	8	0.7%	0.995
Diagnosis of other fungal infection	33	0.2%	42%	14	1.3%	19	1.8%	0.869
Total	2129	11%	50%	1059	–	1070	–	–

In multivariable analysis, male sex, hematological malignancy, influenza, solid organ transplant, and ECMO for respiratory support were associated with an increased risk of *Aspergillus* involvement. Increased weight, nonviral pneumonia, aspiration pneumonitis, ARDS, and acute respiratory failure were associated with a decreased risk of *Aspergillus* involvement (Table 3). With bootstrapping, the associations with hematological malignancies and ARDS were not statistically significant (*see* Additional file 1: Appendix 5). Although steroid administration was positively associated with *Aspergillus* involvement in univariate analysis (OR 1.50, 95% CI 1.07–2.10, *p* = 0.017), this association did not reach statistical significance in the multivariable backward selection model. There was no significant association between *Aspergillus* involvement and chronic pulmonary condition, tobacco use, tuberculosis, or human immunodeficiency virus (HIV).

**Table 3:** Risk factors for *Aspergillus* colonization or infection

Variable	OR	95% CI	*p* Value
Male sex	1.55	1.16–2.08	0.003
Weight/10 kg	0.87	0.82–0.93	< 0.001
Hematological malignancy	2.18	0.99–4.78	0.052
Aspiration pneumonitis	0.33	0.12–0.93	0.036
ARDS	0.75	0.50–1.12	0.154
Acute respiratory failure NOS	0.58	0.42–0.79	0.001

*ARDS* Acute respiratory distress syndrome, *NOS* Not otherwise specified

Advanced age, increased weight, sepsis, and renal replacement therapy (RRT) were independent risk factors for CBSI. Although increased weight, nonviral pneumonia, intra-aortic balloon pump, and cardiopulmonary bypass were associated with the risk of CBSI in the backward selection model, the association could not be validated by bootstrapping (*p* > 0.05) (Table 4; *see* also Additional file 1: Appendix 6]. Diabetes, steroids, femoral cannulation, laparotomy, and pancreatitis were not significantly associated with CBSI. The registry does not collect information on antibiotic or parenteral nutrition use.

**Table 4:** Risk factors for *Candida* bloodstream infection

Variable	OR	95% CI	*p* Value
Age/10 years	1.13	1.03–1.25	0.011
Weight/10 kg	1.05	1.00–1.10	0.061
Sepsis	1.60	1.15–2.23	0.005
Renal replacement therapy	1.55	1.12–2.13	0.007
Nonviral pneumonia	0.70	0.48–1.03	0.068
Intra-aortic balloon pump	1.46	0.94–2.26	0.091
Cardiopulmonary bypass	0.56	0.28–1.15	0.112

As hypothesized, *Aspergillus* involvement (OR 0.40, *p* < 0.001) and CBSI (OR 0.47, *p* < 0.001) were both independently associated with decreased survival. The other factors associated with worse survival in multivariable analysis were advanced age, hematological malignancy, acute kidney injury (AKI), HIV, ARDS, sepsis, nitric oxide, ECMO for cardiac support, and extracorporeal cardiopulmonary resuscitation. However, increased weight, aspiration pneumonitis, influenza, solid organ transplant, nonviral pneumonia, and neuromuscular blockers were independently associated with increased survival in our cohort (Table 5). The associations with AKI, sepsis, and solid organ transplant were not statistically significant with bootstrapping (*see also* Additional file 1: Appendix 7).

**Table 5:** Independent predictors of survival

Variable	OR	95% CI	*p* Value
Age/10 years	0.81	0.77–0.87	< 0.001
Weight/10 kg	1.04	1.01–1.08	0.018
Hematological malignancy	0.44	0.21–0.95	0.035
Acute kidney injury	0.83	0.66–1.05	0.116
HIV infection	0.09	0.02–0.42	0.002
Aspiration pneumonitis	1.87	1.14–3.09	0.014
ARDS	0.66	0.50–0.88	0.004
Influenza	1.67	1.26–2.22	< 0.001
Sepsis	0.80	0.63–1.02	0.07
Solid organ transplant	1.37	0.96–1.96	0.086
Nonviral pneumonia	1.33	1.05–1.68	0.02
Nitric oxide	0.55	0.42–0.73	< 0.001
Neuromuscular blockers	1.26	1.03–1.55	0.027
Cardiac support	0.59	0.47–0.75	< 0.001
ECPR	0.45	0.30–0.67	< 0.001
*Candida* bloodstream infection	0.47	0.34–0.63	< 0.001
*Aspergillus* colonization or infection	0.40	0.30–0.54	< 0.001

*Abbreviations: ARDS* Acute respiratory distress syndrome, *HIV* Human immunodeficiency virus, *ECPR* Extracorporeal cardiopulmonary resuscitation

## Discussion

We found a 10.8% prevalence of fungal infection or colonization in the ELSO registry, including 1.3% of patients with *Aspergillus* involvement and 1.2% of patients with CBSI. These prevalence rates are lower than expected in a population of mechanically ventilated critically ill patients with indwelling catheters and an ICU length of stay generally exceeding 1 week. *Aspergillus* involvement was associated with classic factors that cause immunosuppression, such as hematological malignancy and solid organ transplant, as well as with influenza and ECMO for respiratory support. Not surprisingly, CBSI was associated with typical risk factors such as sepsis and RRT. The survival of patients with any *Aspergillus* involvement (37.5%) was significantly lower than that of the rest of the ELSO population, even after adjusting for other predictive factors in multivariable analysis (OR 0.41). Similarly, CBSI was associated with poor survival (35.9%) independently (OR 0.47).

The prevalence of fungus-positive samples in general ICU patients with a length of stay more than 7 days has been reported to be as high as 59.7% with systematic sampling, much higher than in our cohort [19]. However, cultures reported in the ELSO registry are performed at the discretion of clinicians rather than systematically. It is highly probable that fungi were simply undetected in a significant number of patients. This could have resulted in a significant underestimation of the overall prevalence of fungal colonization and infection as well as of the prevalence of specific infections. Moreover, information about antifungal agent administration was not available. A more liberal use of antifungal prophylaxis in this very sick population of patients could also have contributed to a lower rate of fungus-positive samples.

The overall prevalence of any microbiological or histological evidence of *Aspergillus* in a medical ICU has been reported to be 6.9%, and a combined prevalence of proven, probable, or possible IA has been reported to be 5.8% [14]. These data are more in line with the 7.2% prevalence found in an Australian ICU in a review of their ECMO cases between 2005 and 2011 [20]. There was a much lower prevalence of *Aspergillus* involvement in the ELSO registry. The first caveat in interpreting the rates of *Aspergillus* involvement is that microscopic examination and culture of respiratory tract specimens have a combined sensitivity of only 50% for IA [21]. Second, discriminating between colonization and infection is always challenging without histology. Biopsies are rarely performed in ECMO patients, because they are at high risk of bleeding owing to systemic anticoagulation, platelet and coagulation factor consumption, and increased fibrinolysis associated with the extracorporeal circuit. Nonneutropenic patients most often do not display the characteristic radiological signs of IA [22]. Furthermore, many patients had respiratory failure with already abnormal chest imaging results before developing IA. Newer tests such as galactomannan and PCR of serum or bronchoalveolar lavage specimens have been developed to improve the diagnostic yield for IA [23]. However, results of such tests, if performed, were not collected in the database. Blot and colleagues have described and validated an algorithm for use in diagnosing aspergillosis in critically ill individuals [24]. However, the ELSO registry did not collect all the variables needed to apply their definitions. In addition, whether *Aspergillus* isolated in the lower respiratory tract of critically ill patients can be viewed as a contaminant or colonizer remains a matter of debate [25, 26].

Meersseman and colleagues reported an overall survival of 20% in patients without hematological malignancy with evidence of *Aspergillus* involvement [14]. The higher survival that we found could be due to the fact that ECMO patients, although acutely very ill, are younger and have fewer comorbidities than the general ICU population. Patients with *Aspergillus* involvement in the ELSO registry had a mean age of 46 years, and only 8.5% of them had chronic pulmonary disease, as compared with a mean age of 61 years and 42% with chronic pulmonary disease in the Meersseman study.

The strong association between influenza and *Aspergillus* found in our cohort has been described before in case series and case-control studies. Coinfection rates ranging from 29% to 75% have been reported [27, 28]. Proposed mechanisms for this increased susceptibility to *Aspergillus* invasion include both local and systemic effects [29]. Indeed, influenza induces tracheitis and bronchitis and impairs normal ciliary function [30]. The virus also impairs phagocytosis and induces anti-inflammatory cytokine production leading to T-cell dysfunction and apoptosis [31–33].

In recent large multicentric cohort studies of unselected patients admitted to mixed ICUs, the prevalence of candidemia ranged from 0.33% to 0.69% [3, 34]. Although the rate we found was higher than the one reported in these large studies, in a cohort of patients with severe critical illness and a long ICU length of stay, we expected a much higher rate. A prevalence of 3.3% has been reported in patients with a length of stay in the ICU more than 7 days [19]. Moreover, a prevalence of 6.4% of CBSI was found in a retrospective review of ECMO cases in an Australian center [17]. In keeping with these findings, yeast DNA has been detected in 7% of patients on oxygenators [18]. In theory, this should translate to positive fungal cultures at some point. The discrepancy could be explained by undersampling or a more liberal use of antifungal prophylaxis.

In contrast, CBSI survival was strikingly worse than previously described in the literature. León and colleagues found an overall survival of 43.4% in critically ill patients with invasive *Candida* infection hospitalized for more than 7 days [19]. Others have even found a survival of 57–60% in general ICU cohorts [3, 34]. Patients on ECMO could simply have a higher illness severity than these cohorts. It is also possible that the presence of foreign material makes it more difficult to eradicate *Candida*, as seen with prosthetic valve endocarditis.

Multiple hospitals across the globe in very different settings participate in the ELSO registry. These centers collect data on all their ECMO cases. This confers strong external validity to our study. However, the internal validity of the study is affected by multiple factors partly inherent to its retrospective nature. For instance, microbiological sampling was nonsystematic. This may have caused underestimation of the prevalence of fungal infections. This also weakened the analysis of risk factors by making them highly susceptible to observer bias. Indeed, fungal cultures may have been performed more often in patients with presenting factors that were suspected by clinicians to increase the risk of fungal infection. Moreover, limited data were available on potential confounding factors such as antifungal therapy and prophylaxis. This may have decreased certain associations with risk factors if antifungal therapy was prescribed more often in those patients. Such limitations could be avoided or minimized in a prospective study.

## Conclusions

Patients on ECMO do not seem to develop fungal colonization or infection more frequently than other critically ill patients. *Aspergillus* involvement and CBSI were independently associated with decreased survival. CBSI mortality was higher than described in the general ICU population. *Aspergillus* involvement was associated with respiratory ECMO and influenza. Clinicians should maintain a high index of suspicion in this subgroup, and efforts should be made to establish an early diagnosis. It remains unclear, however, if treating the fungus would improve survival.

## Additional file

Additional file 1: **Appendix 1.** ELSO Registry Case Report Form (document). **Appendix 2.** Patient selection (list). **Appendix 3.** Details of *Aspergillus* involvement (table). **Appendix 4.** Survival of patients according to number of cultures other than blood positive for *Candida* (table). **Appendix 5.** Multiple logistic regression for *Aspergillus* (table). **Appendix 6.** Multiple logistic regression for *Candida* bloodstream infection (table). **Appendix 7.** Multiple logistic regression for survival (table). **Appendix 8.** Case distribution by year (figure). (PDF 1090 kb)

## Abbreviations

AKI: Acute kidney injury
ARDS: Acute respiratory distress syndrome
CBSI: *Candida* bloodstream infection
ECMO: Extracorporeal membrane oxygenation
ECPR: Extracorporeal cardiopulmonary resuscitation
ELSO: Extracorporeal Life Support Organization
HIV: Human immunodeficiency virus
IA: Invasive aspergillosis
ICU: Intensive care unit
RRT: Renal replacement therapy
