# Supplementary material for: Fungal infections in adult patients on extracorporeal life support
Source: Crit Care. 2018 Apr 17;22:98. doi: 10.1186/s13054-018-2023-z (PMC5905180; doi:10.1186/s13054-018-2023-z)
Supplement: Supplementary file 1 — Appendix 1. ELSO Registry Case Report Form (document). Appendix 2. Patient selection (list). Appendix 3. Details of Aspergillus involvement (table). Appendix 4. Survival of patients according to number of cultures other than blood positive for Candida (table). Appendix 5. Multiple logistic regression for Aspergillus (table). Appendix 6. Multiple logistic regression for Candida bloodstream infection (table). Appendix 7. Multiple logistic regression for survival (table). Appendix 8. Case distribution by year (figure). (PDF 1090 kb) [file 13054_2018_2023_MOESM1_ESM.pdf]

APPENDIX 1 – ELSO Registry Case Report Form

ECLS Registry Form
Extracorporeal Life Support Organization (ELSO)

Center ID:______ Center name: ______________________________________ Run No _____
(for this patient)

Unique ID: _______________________ Birth Date/Time _______________________________
Month d, yyyy hh:mm

Sex: _______ (M, F) Race: ________________ (Asian, Black, Hispanic, White, Other)

Neonatal patients:

Birth weight: _____________ kg Maternal age: _________________

Apgar (1 min): ____________ Apgar (5 min): ________________

Delivery: ☐ Vaginal ☐ C-sect ☐ Elective C-sect

Gestational age: ________

CDH present? ☐ (Check if yes) If Yes: Prenatal diagnosis? ☐ (Check if yes)

Side of CDH: ___ (R,L,B) Repair: ☐ None ☐ Pre-ECLS ☐ On ECLS ☐ Post-ECLS

Infant Transported? ☐ (Check if yes) Cardiac arrest at birth? ☐ (Check if yes)

Diagnoses and Procedures

ICD-9 Codes

Primary diagnosis: ____________

Use ICD-9 codes for diagnoses (see diagnosis codes sheet)

Secondary diagnoses: ____________ ____________ ____________
(unlimited) ____________ ____________ ____________

CPT Code **Date/Time of Procedure**

Procedures: ____________ ____________
(unlimited) ____________ ____________
____________ ____________

Use CPT codes for procedures (see procedure codes sheet)

(List all relevant procedures related to the patient even if preceding this admission)

Cultured Organisms:
(unlimited)

Organism: ______________ Site:________________ Date/Time __________________
Organism: ______________ Site:________________ Date/Time __________________
Organism: ______________ Site:________________ Date/Time __________________
Organism: ______________ Site:________________ Date/Time __________________

Sites: Blood, Bone, Cerebrospinal fluid, Peritoneal fluid, Pleural fluid, Respiratory tract, Skin/soft tissue, Stool, Urine, Wound – surgical, Wound – traumatic, Other, Unknown

Pre-ECLS Course

Date / Time admitted: ____________________________ Intubated: ____________________________
(to ECLS center) Month d, yyyy hh:mm Month d, yyyy hh:mm

Current weight (kg): ___________

Patient Transported: ☐ Pre-ECLS cardiac arrest: ☐ Bridge to transplant: ☐

Pre-ECLS support: ________ ________ ________ ________ ________ (Use Support Codes)

________ ________ ________ ________ ________

Pre-ECLS blood gases:

Date/Time: ___________________________ (Worst in last 6 hours)

pH: _________ PaCO2: ________ PaO2:_________ HCO3: ________ SaO2(%):_________

Pre-ECLS ventilator settings:

Date/Time:___________________________ (Worst in last 6 hours)

Rate/Hz: _______ FiO2(%):_______ PIP/Ampl: _____ PEEP: _______ MAP: ________

Hand bagging: ☐ (check if yes) Vent type: ☐ Conventional ☐ HFO ☐ Other HFV

Pre-ECLS hemodynamics:

Date/Time: ___________________________ (Worst in last 6 hours)

BP: ________ (Systolic) ________ (Diastolic) ________ (Mean) SvO_2: __________ PCWP: ________

PAP: ________ (Systolic) ________ (Diastolic) ________ (Mean) CI: ____________

ECLS Course

Date/Time on ECLS: _________________________ Date/Time off ECLS: _____________________
Month d, yyyy hh:mm Month d, yyyy hh:mm

Reason for support: ☐ Pulmonary ☐ Cardiac ☐ ECPR

ECLS mode: ____________________________________
(VA, VA+V, VA-VV, VV, VV-VA, VVA, VVDL, VVDL+V, Other)

Cannulations:

Site	Percutaneous	Manufacturer	Size (Fr)	Length (cm)
____	☐ Yes	____________________	_____	_____
____	☐ Yes	____________________	_____	_____
____	☐ Yes	____________________	_____	_____

Site: RCCA, LCCA, RIJV, RIJVC, LIJV, RFA, LFA, RFV, LFV, RA, LA, PA, Aorta, Other

Equipment used:

Membrane lung: ______________________________

Heat exchanger: ______________________________

Pump: ______________________________

Hemofilter: ______________________________

Pump flow: Units: ☐ ml/kg/min ☐ ml/min ☐ L/min (Check one)

4th hour flow: __________ 24th hour: ____________

24 hour ECLS blood gases:

Date/Time: ______________________ (Best values at 24 hours of ECLS)

pH: ________ PaCO2: ________ PaO2:________ HCO3: ________ SaO2(%):________

24 hour ECLS ventilator settings:

Date/Time: ______________________ (Best values at 24 hours of ECLS)

Rate/Hz: ______ FiO2(%):_______ PIP/Ampl: _____ PEEP: ______ MAP: _______

Hand bagging: ☐ (check if yes) Vent type: ☐ Conventional ☐ HFO ☐ Other HFV

24 hour hemodynamics:

Date/Time: ______________________ (Best values at 24 hours of ECLS)

BP: ________ (Systolic) ________ (Diastolic) ________ (Mean) SvO2: ________ PCWP: _______

PAP: ________ (Systolic) ________ (Diastolic) ________ (Mean) CI: __________

ECLS Complications

Mechanical complications: (requiring change of equipment or intervention)

- ☐ Oxygenator failure
- ☐ Raceway rupture
- ☐ Other tubing rupture
- ☐ Pump malfunction
- ☐ Heat exch. Malfunction
- ☐ Clots: oxygenator
- ☐ Clots: bridge
- ☐ Clots: bladder
- ☐ Clots: hemofilter
- ☐ Clots: other
- ☐ Air in circuit
- ☐ Cracks: connectors
- ☐ Cannula problems

Patient complications:

Hemorrhagic complications: (requiring transfusion or other intervention)

- ☐ GI hemorrhage
- ☐ Cannulation site bleeding
- ☐ Surgical site bleeding
- ☐ Hemolysis (plasma hgb > 50 mg/dl)
- ☐ DIC

Neurologic complications:

- ☐ Clinical brain death
- ☐ Clinical seizures
- ☐ EEG seizures
- ☐ Infarction (US or CT)
- ☐ Hemorrhage (US or CT)

Renal complications:

- ☐ Creatinine 1.5-3.0
- ☐ Creatinine > 3.0
- ☐ Hemodialysis
- ☐ Hemofiltration
- ☐ CAVHD

Pulmonary complications: (requiring intervention)

- ☐ Pneumothorax
- ☐ Pulmonary hemorrhage

Cardiopulmonary complications: (requiring intervention)

- ☐ Inotropes on ECLS
- ☐ CPR required
- ☐ Myocardial stun by echocardiography
- ☐ Cardiac arrhythmia
- ☐ Hypertension requiring vasodilator treatment
- ☐ PDA: R→L
- ☐ PDA: L→R
- ☐ PDA: Bidirectional
- ☐ PDA: Unknown
- ☐ Tamponade: Blood
- ☐ Tamponade: Serous
- ☐ Tamponade: Air

Infectious complications:

- ☐ Culture proven new infection (code organism under Diagnosis and Procedures)
- ☐ WBC < 1,500

Metabolic complications:

- ☐ Glucose < 40
- ☐ Glucose > 240
- ☐ pH < 7.20
- ☐ pH > 7.60
- ☐ Hyperbilirubinemia (> 2 direct, > 13 indirect, or > 15 total)

Outcome

Reason for discontinuing ECLS: ☐ Recovery (survived ECMO) ☐ Died on ECLS, or ECLS withdrawn in anticipation of death

If died on ECLS or ECLS withdrawn in anticipation of death select 1 reason:

- ☐ Parental or family request
- ☐ Hemorrhage
- ☐ Diagnosis incompatible with life
- ☐ Irreversible organ failure: (Select all that apply)
  - ☐ CNS ☐ Pulmonary ☐ Cardiac
  - ☐ Liver ☐ Infection ☐ Other

Cannula site repair: ☐None ☐ Carotid artery ☐ Jugular vein ☐ Both ☐ Other

Date / time of final extubation: ____________________________

☐ Discharged alive (from ECLS center)

Date / time of discharge / transfer: ______________________________

Discharge location: ☐ Home ☐ In-hospital service ☐ Referral hospital ☐ Other facility (hospital or rehab facility)

Date / time of death: ______________________________

If death, code primary cause of death and contributing causes under Diagnosis and Procedures

Form completed by: __________________________ Date completed: ________________

ELSO Office, 2800 Plymouth Rd, Building 300, Room 303, Ann Arbor, MI 48109-2800 Phone: 734-998-6601

APPENDIX 2 – Patient Selection

- ICD9 diagnosis

  Primary or Secondary diagnosis < 1 day after the initiation of ECMO:

  112.4 Candidal pneumonia

  112.5 Systemic candidiasis

  112.81 Candidal endocarditis

  112.83 Candidal meningitis

  114.0 Primary coccidioidomycosis (pulmonary)

  115* Histoplasmosis

  116* Blastomycotic infection

  117.1 Sporotrichosis

  117.3 Aspergillosis

  117.5 Cryptococcosis

  117.6 Allescheriosis [Petriellidosis]

  117.7 Zygomycosis [Phycomycosis or Mucormycosis]

  117.8 Infection by dematiacious fungi [Phaehyphomycosis]

  117.9 Other and unspecified mycoses

  484.6 Pneumonia in aspergillosis

- Organisms ID:

50	*Aspergillus fumigatus*
51	*Aspergillus sp.*
21	*Candida albicans*
81	*Candida krusei*
53	*Candida parapsilosis*
99	*Candida sp.*
82	*Candida tropicalis*
41	Yeast sp.

APPENDIX 3 – Details of *Aspergillus* involvement

	**n**
Aspergillosis Diagnosis	51
Aspergillus Pneumonia Diagnosis	24
AspergillusBloodCult	29
AspergillusRespCult	200
AspergillusOtherCult	36

APPENDIX 4 – Survival of patients according to number of cultures other than blood positive for *Candida*

Number of cultures	Survival
1	53.1%
2	52.3%
3	55.0%

APPENDIX 5 – Multiple Logistic Regression for *Aspergillus*

	Variable	B	S.E.	Wald	df	Sig.	Exp(B)
Step 1	Age / 10 years	-0.015	0.046	0.111	1	0.739	0.985
	Male gender	0.465	0.153	9.234	1	0.002	1.592
	Weight	-0.141	0.033	18.488	1	0	0.869
	Tobacco	0.365	0.367	0.988	1	0.32	1.441
	Chronic Pulmonary Disease	-0.155	0.259	0.358	1	0.55	0.856
	Diabetes	0.14	0.258	0.295	1	0.587	1.15
	CKD	-0.411	0.492	0.7	1	0.403	0.663
	Chronic Liver Disease	0.703	0.557	1.59	1	0.207	2.019
	Hematological malignancy	0.745	0.406	3.372	1	0.066	2.107
	Non-hematological malignancy	0.021	0.404	0.003	1	0.959	1.021
	AKI	-0.158	0.195	0.652	1	0.419	0.854
	Stroke	0.202	0.58	0.121	1	0.728	1.223
	Pancreatitis	-0.937	1.04	0.81	1	0.368	0.392
	Acute Liver Disease	0.425	0.427	0.992	1	0.319	1.53
	Tuberculosis	-1.184	1.076	1.209	1	0.271	0.306
	HIV	-0.592	0.778	0.579	1	0.447	0.553
	Viral Pneumonia	0.146	0.24	0.372	1	0.542	1.157
	Aspiration Pneumonitis	-1.144	0.531	4.65	1	0.031	0.318
	ARDS	-0.322	0.211	2.345	1	0.126	0.724
	Acute Resp Failure NOS	-0.551	0.168	10.763	1	0.001	0.576
	Cardiac Arrest	-0.279	0.331	0.712	1	0.399	0.756
	Inotropes/vasopressors	0.033	0.164	0.04	1	0.842	1.033
	Steroids	0.242	0.191	1.596	1	0.207	1.274
	Plasmapheresis	0.837	0.825	1.028	1	0.311	2.309
	Influenza	0.879	0.183	23.046	1	0	2.408
	Sepsis	0.096	0.179	0.289	1	0.591	1.101
	Solid Organ Transplant	0.581	0.235	6.123	1	0.013	1.788
	Renal Replacement Therapy	0.032	0.179	0.031	1	0.86	1.032

	Non-viral Pneumonia	-0.411	0.184	5.005	1	0.025	0.663
	Respiratory Support	0.918	0.225	16.607	1	0	2.504
	Heart Failure	-0.127	0.223	0.324	1	0.569	0.881
	Constant	-1.616	0.401	16.242	1	0	0.199
Step 22	Male gender	0.441	0.148	8.901	1	0.003	1.554
	Weight / 10kg	-0.136	0.032	18.215	1	0	0.873
	Hematological malignancy	0.778	0.401	3.765	1	0.052	2.178
	Aspiration Pneumonitis	-1.099	0.524	4.393	1	0.036	0.333
	ARDS	-0.29	0.204	2.028	1	0.154	0.748
	Acute Resp Failure NOS	-0.548	0.16	11.708	1	0.001	0.578
	Influenza	0.909	0.175	26.887	1	0	2.482
	Solid Organ Transplant	0.605	0.228	7.031	1	0.008	1.831
	Non Viral Pneumonia	-0.37	0.176	4.4	1	0.036	0.691
	Respiratory Support	1.013	0.184	30.442	1	0	2.755
	Constant	-1.762	0.293	36.112	1	0	0.172

Bootstrap Aspergillus Model

**Variable**	**OR**	**CI.95**	**p-value**
Male gender	1.55	1.13-2.15	0.003
Weight / 10kg	0.87	0.82-0.92	0.001
Hematological malignancy	2.18	0.81-5.05	0.056
Aspiration Pneumonitis	0.33	0.08-0.70	0.022
ARDS	0.75	0.52-1.04	0.152
Acute Respiratory Failure NOS	0.58	0.41-0.75	0.001
Influenza	2.48	1.76-3.50	0.001
Solid Organ Transplant	1.83	1.09-2.79	0.010
Non-Viral Pneumonia	0.69	0.46-0.97	0.040
Respiratory Support	2.75	1.92-3.93	0.001

APPENDIX 6 – Multiple Logistic Regression for *Candida* bloodstream infection

	Variable	B	S.E.	Wald	df	Sig.	Exp(B)
Step 1	Age / 10 years	0.118	0.051	5.319	1	0.021	1.125
	Male	0.115	0.162	0.5	1	0.479	1.122
	Weight / 10 kg	0.055	0.027	4.059	1	0.044	1.057
	Tobacco	-0.555	0.436	1.617	1	0.203	0.574
	Chronic Pulmonary Disease	0.068	0.281	0.058	1	0.81	1.07
	Diabetes	-0.376	0.274	1.876	1	0.171	0.687
	CKD	0.46	0.35	1.726	1	0.189	1.583
	Chronic Liver Disease	-0.112	0.652	0.029	1	0.864	0.894
	Hematological malignancy	0.165	0.555	0.089	1	0.766	1.18
	Non-hematological malignancy	-0.136	0.427	0.101	1	0.751	0.873
	AKI	0.011	0.197	0.003	1	0.954	1.012
	Stroke	-0.293	0.633	0.215	1	0.643	0.746
	Pancreatitis	0.789	0.591	1.786	1	0.181	2.202
	Acute Liver Disease	0.282	0.366	0.591	1	0.442	1.325
	Tuberculosis	0.445	0.793	0.314	1	0.575	1.56
	Viral Pneumonia	-0.006	0.312	0	1	0.984	0.994
	Aspiration Pneumonitis	-0.269	0.423	0.404	1	0.525	0.764
	ARDS	-0.279	0.243	1.312	1	0.252	0.757
	Acute Resp Failure NOS	-0.052	0.173	0.089	1	0.766	0.95
	Cardiac Arrest	-0.123	0.276	0.199	1	0.656	0.884
	Inotropes/vasopressors	0.055	0.187	0.086	1	0.77	1.056
	Steroids	-0.023	0.223	0.011	1	0.917	0.977
	Plasmapheresis	-0.082	1.08	0.006	1	0.939	0.921
	Influenza	-0.118	0.239	0.245	1	0.621	0.888
	Sepsis	0.532	0.179	8.815	1	0.003	1.702
	Solid Organ Transplant	0.205	0.279	0.538	1	0.463	1.228
	Renal Replacement Therapy	0.407	0.178	5.231	1	0.022	1.502
	Non-viral Pneumonia	-0.305	0.201	2.313	1	0.128	0.737
	Respiratory Support	-0.225	0.224	1.014	1	0.314	0.798
	Heart Failure	-0.196	0.211	0.862	1	0.353	0.822
	Femoral cannula	-0.049	0.167	0.088	1	0.767	0.952
	IABP	0.309	0.25	1.531	1	0.216	1.362
	VAD	0.27	0.395	0.469	1	0.493	1.31
	CBP	-0.672	0.372	3.257	1	0.071	0.511

	Laparotomy	0.378	0.358	1.116	1	0.291	1.459
	Constant	-3.139	0.448	49.012	1	0	0.043
Step 29	Age /10 years	0.124	0.049	6.509	1	0.011	1.132
	Weight / 10 years	0.048	0.026	3.501	1	0.061	1.049
	Sepsis	0.471	0.168	7.847	1	0.005	1.602
	Renal Replacement Therapy	0.437	0.163	7.186	1	0.007	1.548
	Non Viral Pneumonia	-0.352	0.193	3.321	1	0.068	0.704
	Intra-Aortic Balloon Pump	0.377	0.223	2.865	1	0.091	1.458
	Cardiopulmonary Bypass	-0.576	0.363	2.522	1	0.112	0.562
	Constant	-3.29	0.355	85.76	1	0	0.037

Bootstrap CBSI model

**Variable**	**OR**	**CI.95**	**p-value**
Age /10 years	1.13	1.03-1.24	0.005
Weight / 10 years	1.04	0.98-1.10	0.122
Sepsis	1.64	1.17-2.34	0.001
Renal Replacement Therapy	1.49	1.04-2.03	0.010
Non-Viral Pneumonia	0.77	0.54-1.06	0.166
Intra-Aortic Balloon Pump	1.43	0.84-2.23	0.111
Cardiopulmonary Bypass	0.62	0.27-1.09	0.142

APPENDIX 7 – Multiple Logistic Regression for Survival

	Variable	B	S.E.	Wald	df	Sig.	Exp(B)
Step 1	Age	-0.202	0.032	39.514	1	0	0.817
	Male	-0.083	0.102	0.659	1	0.417	0.92
	Weight	0.045	0.019	5.518	1	0.019	1.046
	Tobacco	0.352	0.251	1.958	1	0.162	1.421
	Chronic Pulmonary Disease	-0.098	0.178	0.305	1	0.581	0.906
	Diabetes	0.224	0.167	1.788	1	0.181	1.251
	CKD	0.027	0.267	0.01	1	0.92	1.027
	Chronic Liver Disease	-0.499	0.447	1.244	1	0.265	0.607
	Hematological malignancy	-0.819	0.39	4.415	1	0.036	0.441
	Non-hematological malignancy	-0.218	0.284	0.593	1	0.441	0.804
	AKI	-0.129	0.13	0.975	1	0.323	0.879
	Stroke	-0.507	0.393	1.667	1	0.197	0.602
	Pancreatitis	0.056	0.469	0.014	1	0.906	1.057
	Acute Liver Disease	-0.271	0.267	1.029	1	0.31	0.763
	Tuberculosis	0.321	0.58	0.306	1	0.58	1.378
	HIV	-2.327	0.777	8.97	1	0.003	0.098
	Viral Pneumonia	-0.125	0.194	0.415	1	0.52	0.883
	Aspiration Pneumonitis	0.686	0.262	6.856	1	0.009	1.987
	ARDS	-0.387	0.148	6.865	1	0.009	0.679
	Acute Resp Failure NOS	0.012	0.11	0.011	1	0.917	1.012
	Cardiac Arrest	-0.033	0.188	0.031	1	0.861	0.968
	Inotrope/vasopressor	-0.074	0.115	0.413	1	0.52	0.929
	Steroids	-0.113	0.148	0.583	1	0.445	0.893
	Plasmapheresis	-0.269	0.65	0.171	1	0.68	0.765
	Influenza	0.481	0.15	10.321	1	0.001	1.617
	Sepsis	-0.198	0.124	2.523	1	0.112	0.821
	Solid Organ Transplant	0.318	0.189	2.85	1	0.091	1.375
	Renal Replacement Therapy	-0.009	0.124	0.005	1	0.944	0.991
	Non-viral Pneumonia	0.278	0.124	5.033	1	0.025	1.32
	Heart Failure	-0.053	0.139	0.147	1	0.702	0.948

	IABP	-0.173	0.174	0.989	1	0.32	0.841
	VAD	-0.236	0.298	0.628	1	0.428	0.79
	CBP	0.198	0.211	0.886	1	0.347	1.219
	Laparotomy	-0.376	0.272	1.906	1	0.167	0.687
	Candida Bloodstream Infection	-0.733	0.157	21.791	1	0	0.48
	Aspergillus	-0.89	0.151	34.539	1	0	0.411
	Nitric Oxide	-0.614	0.145	17.934	1	0	0.541
	Epoprostenol	0.081	0.249	0.107	1	0.744	1.085
	NMB	0.257	0.112	5.308	1	0.021	1.293
	Support Type			12.875	2	0.002	
	Cardiac Support	-0.447	0.154	8.447	1	0.004	0.64
	ECPR Support	-0.715	0.234	9.295	1	0.002	0.489
	Constant	1.09	0.247	19.427	1	0	2.975
Step 25	Age /10 years	-0.205	0.031	44.048	1	0	0.814
	Weight / 10 years	0.044	0.018	5.595	1	0.018	1.044
	Hematological malignancy	-0.813	0.387	4.421	1	0.035	0.444
	Acute Kidney Injury	-0.186	0.118	2.474	1	0.116	0.83
	HIV	-2.375	0.772	9.465	1	0.002	0.093
	Aspiration Pneumonitis	0.627	0.255	6.052	1	0.014	1.873
	ARDS	-0.411	0.142	8.366	1	0.004	0.663
	Influenza	0.513	0.145	12.448	1	0	1.67
	Sepsis	-0.219	0.121	3.293	1	0.07	0.803
	Solid Organ Transplant	0.315	0.183	2.947	1	0.086	1.37
	Non-Viral Pneumonia	0.284	0.121	5.453	1	0.02	1.328
	Candida Bloodstream Infection	-0.762	0.155	24.086	1	0	0.467
	Aspergillus colonization or infection	-0.917	0.15	37.487	1	0	0.4
	Nitric Oxide	-0.593	0.142	17.322	1	0	0.553
	Neuromuscular Blockers	0.233	0.105	4.898	1	0.027	1.262
	SupportType			28.082	2	0	
	Cardiac support	-0.52	0.118	19.359	1	0	0.595
	ECPR support	-0.795	0.204	15.204	1	0	0.452
	Constant	1.011	0.229	19.527	1	0	2.75

Bootstrap Survival model

**Variable**	**OR**	**CI.95**	**p-value**
Age /10 years	0.81	0.77-0.86	0.001
Weight / 10 years	1.04	1.00-1.09	0.026
Hematological malignancy	0.43	0.18-0.88	0.038
Acute Kidney Injury	0.82	0.64-1.06	0.108
HIV	0.09	0.00-0.22	0.002
Aspiration Pneumonitis	1.93	1.23-3.23	0.008
ARDS	0.66	0.50-0.87	0.003
Influenza	1.71	1.24-2.35	0.002
Sepsis	0.80	0.63-1.01	0.080
Solid Organ Transplant	1.38	0.95-2.09	0.086
Non-Viral Pneumonia	1.32	1.03-1.69	0.023
Nitric Oxide	0.55	0.43-0.72	0.001
Neuromuscular Blockers	1.27	1.02-1.58	0.022
Cardiac support	0.60	0.48-0.75	0.001
ECPR	0.45	0.29-0.69	0.001
Candida Bloodstream Infection	0.48	0.35-0.63	0.001
Aspergillus colonization or infection	0.40	0.31-0.51	0.001

APPENDIX 8 – Case distribution by year

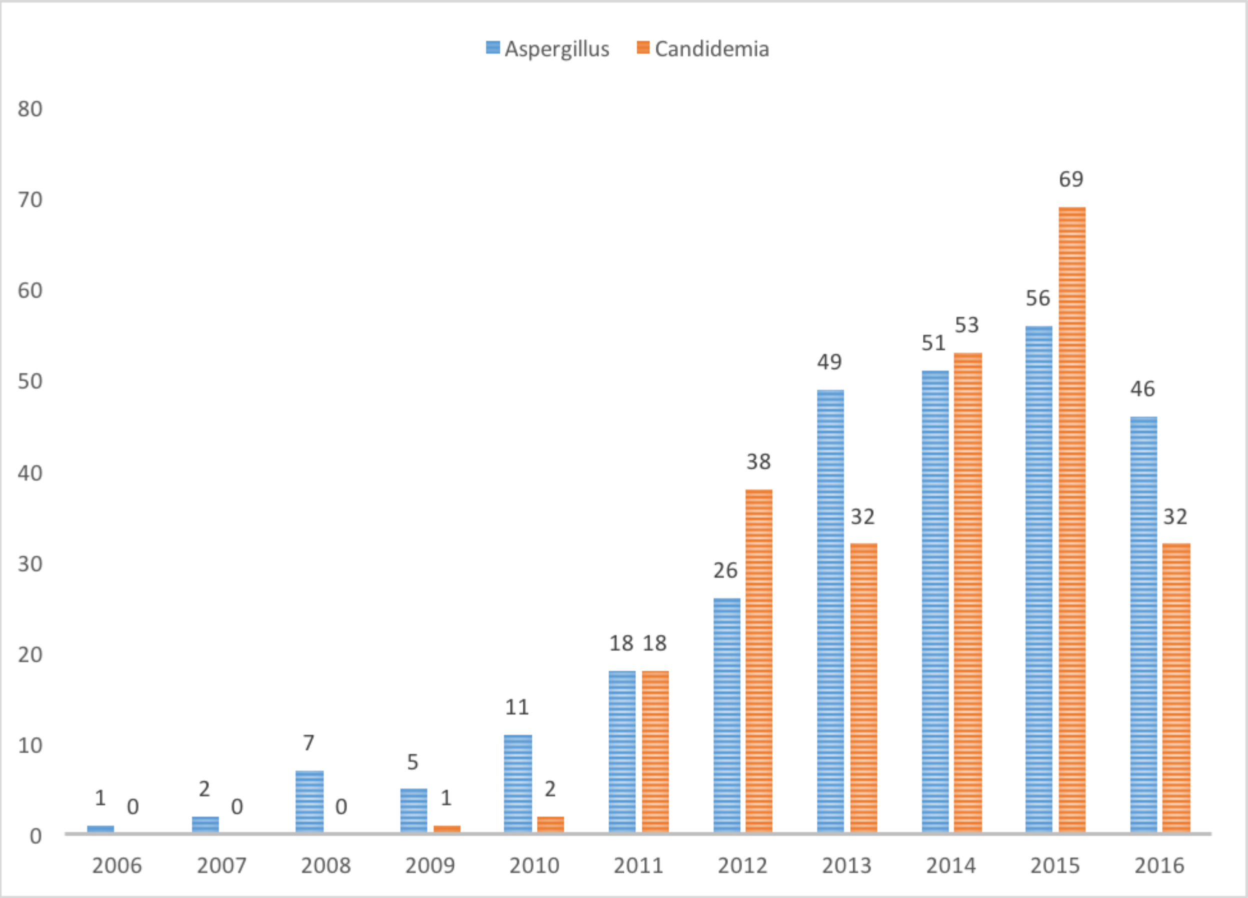

*Please note patients were included up to September 2016.
